# Supplementary material for: Dynamic transcriptomic profiles of zebrafish gills in response to zinc supplementation
Source: BMC Genomics. 2010 Oct 11;11:553. doi: 10.1186/1471-2164-11-553 (PMC3091702; doi:10.1186/1471-2164-11-553)
Supplement: Additional file 2 — Interactive Direct Interaction Network representing the molecular interactions between zinc, copper, iron, calcium and proteins encoded by transcripts changed by zinc supplementation. Mini web-site containing index.html and hyperlinked pages in subdirectory describing a Direct Interaction Network automatically generated based on curated interactions contained within the proprietary PathwayArchitect database. Ovals represent proteins and the circles symbolize metal ions. Objects are coloured by their abundance in zebrafish at the time-point they were significantly different from the control is a scale from -4 fold (dark green) to +4 fold (dark red). Where significant differences were found at more than one time-point, the colour overlay shows expression at the first instance. Dark blue squares denote 'binding', and light blue squares 'expression'; green squares stand for 'regulation', green diamonds for 'metabolism', and green circles for 'promoter binding'. Arrow heads indicate directionality of the interaction where annotated. All nodes and edges can be further interrogated by selecting the relative area of the image. [file 1471-2164-11-553-S2.zip › PathwayArchitect Zn xs DIN/80911.html]

# PROTEIN: INSR

|  |  |
| --- | --- |
| Name | INSR |
| Type | PROTEIN |
| Description | insulin receptor |
| Note | After removal of the precursor signal peptide, the insulin receptor precursor is post-translationally cleaved into two chains (alpha and beta) that are covalently linked. Binding of insulin to the insulin receptor (INSR) stimulates glucose uptake. |
| Alias | INSR |
|  | Insr |
|  | CD220 |
|  | CD220 antigen |
|  | 4932439J01Rik |
|  | IR |


---

|  |  |
| --- | --- |
| GO Component | integral to membrane |
|  | plasma membrane |
|  | integral to plasma membrane |
|  | membrane |


---

|  |  |
| --- | --- |
| GO ID | GO:0016020 |
|  | GO:0005524 |
|  | GO:0008286 |
|  | GO:0030238 |
|  | GO:0004716 |
|  | GO:0004713 |
|  | GO:0007169 |
|  | GO:0005515 |
|  | GO:0005887 |
|  | GO:0000166 |
|  | GO:0004672 |
|  | GO:0005975 |
|  | GO:0016021 |
|  | GO:0005886 |
|  | GO:0004714 |
|  | GO:0007165 |
|  | GO:0004872 |
|  | GO:0016301 |
|  | GO:0006091 |
|  | GO:0006468 |
|  | GO:0009887 |
|  | GO:0005006 |
|  | GO:0007275 |
|  | GO:0005066 |
|  | GO:0016740 |


---

|  |  |
| --- | --- |
| MIM | MIM:147670 |
|  | MIM:262190 |
|  | MIM:246200 |


---

|  |  |
| --- | --- |
| Connectivity | 2637 |


---

|  |  |
| --- | --- |
| Entrez ID | 3643 |
|  | 24954 |
|  | 16337 |


---

|  |  |
| --- | --- |
| Agilent ID | A\_14\_P118054 |
|  | A\_52\_P6490 |
|  | A\_14\_P137289 |
|  | A\_14\_P119488 |
|  | A\_53\_P157252 |
|  | A\_14\_P122948 |
|  | A\_53\_P134492 |
|  | A\_51\_P194843 |
|  | A\_14\_P105430 |
|  | A\_44\_P179858 |
|  | A\_14\_P134909 |
|  | A\_23\_P4764 |


---

|  |  |
| --- | --- |
| Cellular Localization | Plasma membrane |
|  | Membrane |
|  | Cell |


---

|  |  |
| --- | --- |
| DbXref | KEGG pathway##04930##Type II diabetes mellitus##http://www.genome.jp/dbget-bin/show\_pathway?mmu04930+16337 |
|  | KEGG pathway##05050##Dentatorubropallidoluysian atrophy (DRPLA)##http://www.genome.jp/dbget-bin/show\_pathway?rno05050+24954 |
|  | KEGG pathway##05050##Dentatorubropallidoluysian atrophy (DRPLA)##http://www.genome.jp/dbget-bin/show\_pathway?hsa05050+3643 |
|  | KEGG pathway##05050##Dentatorubropallidoluysian atrophy (DRPLA)##http://www.genome.jp/dbget-bin/show\_pathway?mmu05050+16337 |
|  | KEGG pathway##04520##Adherens junction##http://www.genome.jp/dbget-bin/show\_pathway?rno04520+24954 |
|  | Reactome##74749##Signal attenuation##http://www.reactome.org/cgi-bin/eventbrowser?DB=gk\_current&ID=74749 |
|  | Reactome##172365##Signal attenuation##http://www.reactome.org/cgi-bin/eventbrowser?DB=gk\_current&ID=172365 |
|  | KEGG pathway##04910##Insulin signaling pathway##http://www.genome.jp/dbget-bin/show\_pathway?mmu04910+16337 |
|  | KEGG pathway##04520##Adherens junction##http://www.genome.jp/dbget-bin/show\_pathway?mmu04520+16337 |
|  | Reactome##74752##Insulin receptor mediated signalling##http://www.reactome.org/cgi-bin/eventbrowser?DB=gk\_current&ID=74752 |
|  | KEGG pathway##04520##Adherens junction##http://www.genome.jp/dbget-bin/show\_pathway?hsa04520+3643 |
|  | Reactome##172541##Insulin receptor mediated signalling##http://www.reactome.org/cgi-bin/eventbrowser?DB=gk\_current&ID=172541 |


---

|  |  |
| --- | --- |
| Pathway | Insulin Signaling |
|  | Zn xs inventory |
|  | Zn xs DIN |


---

|  |  |
| --- | --- |
| GO Process | male sex determination |
|  | signal transduction |
|  | generation of precursor metabolites and energy |
|  | transmembrane receptor protein tyrosine kinase signaling pathway |
|  | protein amino acid phosphorylation |
|  | insulin receptor signaling pathway |
|  | carbohydrate metabolism |
|  | organogenesis |
|  | development |
|  | organ morphogenesis |


---

|  |  |
| --- | --- |
| UniGene | Hs.465744 |
|  | Mm.378945 |
|  | Rn.9876 |


---

|  |  |
| --- | --- |
| Affymetrix Probeset ID | 102146\_at |
|  | 1369051\_at |
|  | 1450225\_at |
|  | 1572\_s\_at |
|  | 207851\_s\_at |
|  | 213792\_s\_at |
|  | 226212\_s\_at |
|  | 226216\_at |
|  | 226450\_at |
|  | 227432\_s\_at |
|  | 1421380\_at |
|  | 243002\_at |
|  | 33162\_at |
|  | 47628\_at |
|  | 1392043\_at |
|  | 65110\_at |
|  | 70578\_at |
|  | 70604\_at |
|  | 77743\_at |
|  | 88319\_at |
|  | g4557883\_3p\_a\_at |
|  | Hs.208334.0.S1\_3p\_at |
|  | Hs.208334.0.S2\_3p\_s\_at |
|  | Hs.270101.0.A1\_3p\_at |
|  | Hs.89695.1.A1\_3p\_at |
|  | Hs.89695.2.A1\_3p\_at |
|  | Hs.98401.0.S1\_3p\_at |
|  | M10051\_s\_at |
|  | M29014\_at |
|  | Msa.856.0\_at |
|  | rc\_AI070496\_at |
|  | X02160\_at |
|  | 44163\_at |
|  | 111151\_f\_at |
|  | RC\_AA056206\_at |
|  | RC\_AA437233\_at |
|  | RC\_R05296\_at |
|  | TC31492\_at |
|  | TC31519\_at |


---

|  |  |
| --- | --- |
| EC Number | EC 2.7.1.112 |


---

|  |  |
| --- | --- |
| GO Function | protein-tyrosine kinase activity |
|  | transmembrane receptor protein tyrosine kinase activity |
|  | receptor signaling protein tyrosine kinase activity |
|  | transferase activity |
|  | protein kinase activity |
|  | nucleotide binding |
|  | ATP binding |
|  | transmembrane receptor protein tyrosine kinase signaling protein activity |
|  | protein binding |
|  | epidermal growth factor receptor activity |
|  | receptor activity |
|  | kinase activity |


---

|  |  |
| --- | --- |
| Nucleotide | J03466 |
|  | M29930 |
|  | AF110216 |
|  | AK052187 |
|  | NM\_000208 |
|  | AF110214 |
|  | AF110222 |
|  | M10051 |
|  | AF005777 |
|  | M29929 |
|  | NM\_017071 |
|  | M29014 |
|  | X02160 |
|  | AB208861 |
|  | AF110219 |
|  | M76592 |
|  | AF110201 |
|  | J05043 |
|  | AK164267 |
|  | AF110209 |
|  | BC047591 |
|  | M28869 |
|  | M24555 |
|  | AY566293 |
|  | AF110202 |
|  | AF110213 |
|  | AF110210 |
|  | AF005776 |
|  | J05149 |
|  | AF110206 |
|  | AF110220 |
|  | M32972 |
|  | AF110205 |
|  | DQ021481 |
|  | AF110203 |
|  | NM\_010568 |
|  | AF110211 |
|  | AF110217 |
|  | S76825 |
|  | AF110218 |
|  | AF110200 |
|  | AF110207 |
|  | AF110208 |
|  | AF110212 |
|  | AF110204 |
|  | M27197 |


---

|  |  |
| --- | --- |
| Protein | AAD40886 |
|  | AAA41441 |
|  | P15127 |
|  | AAB61415 |
|  | AAD40892 |
|  | AAD40894 |
|  | AAA59174 |
|  | AAD40891 |
|  | AAD40893 |
|  | AAD40888 |
|  | AAA39318 |
|  | AAA59176 |
|  | AAD40896 |
|  | NP\_058767 |
|  | AAA39319 |
|  | AAA59452 |
|  | CAA26096 |
|  | AAA86791 |
|  | AAA59177 |
|  | AAY44085 |
|  | AAH47591 |
|  | BAE43350 |
|  | AAC37604 |
|  | AAD40897 |
|  | AAA59175 |
|  | NP\_034698 |
|  | AAT00542 |
|  | AAA59190 |
|  | AAB61414 |
|  | AAD40887 |
|  | AAD40895 |
|  | AAA59178 |
|  | AAD40889 |
|  | BAD92098 |
|  | AAB33426 |
|  | AAD40890 |
|  | P06213 |
|  | BAE37711 |
|  | NP\_000199 |
|  | P15208 |


---

|  |  |
| --- | --- |
| Organism | Mammal |


---

|  |  |
| --- | --- |
| Location | chromosome 19, 19p13.3-p13.2 (Homo sapiens) |
|  | 8 1.0 cM (Mus musculus) |
|  | chromosome 8, 8 1.0 cM, 8 A1.1 (Mus musculus) |
|  | chromosome 12, 12q12 (Rattus norvegicus) |


---

|  |  |
| --- | --- |
